# Hedgehog, Chamomile and Multipetal Polymeric Structures on the Nanoparticle Surface: Theoretical Insights

**DOI:** 10.3390/polym14204358

**Published:** 2022-10-16

**Authors:** Aleksandra S. Ushakova, Valentina V. Vasilevskaya

**Affiliations:** 1 A.N. Nesmeyanov Institute of Organoelement Compounds RAS, Vavilova St. 28, 119991 Moscow, Russia; 2 Chemistry Department, M. V. Lomonosov Moscow State University, Leninskie Gory, 119991 Moscow, Russia

**Keywords:** amphiphilic homopolymers, self-assembly, nanoparticles

## Abstract

An analytical theory describing the variety of different morphological structures that spontaneously self-assemble in layers of amphiphilic homopolymers tightly grafted to spherical nanoparticle is proposed. For this purpose, the following structures were identified and outlined: hedgehogs, in which macromolecules are combined into cylindrical aggregates; chamomile, when cylindrical aggregates are connected by their ends into loops; multipetal structure with macromolecules self-assembling into thin lamellae; and unstructured, swollen and uniformly compacted shells. The results are presented in the form of state diagrams and serve as a basis for the directional design of the surface pattern by varying system parameters (particle radius, grafting density and degree of polymerization) and solvent properties (quality and selectivity).

## 1. Introduction

Nanoparticles coated with a polymer layer are actively used to design modern composite materials and find application in divers’ areas from drug delivery [1,2,3] to oil production [4,5].

The grafted macromolecules create a protective shell [6,7,8], preserve nanoparticle aggregation and, at the same time, due to their polymeric nature, they are capable of being structured and responding to external influences [9,10,11,12,13].

An undoubted advantage of such systems is the huge possibility of varying their properties by changing the parameters of the grafted layers, such as the grafting density and polymer length [11,14], regularities in the distribution of grafting points [15,16], combination of different macromolecules [6,10,17,18,19], etc. Additionally, thanks to the modern methods of synthesis and subsequent post-polymerization modification many of these options and most possible combinations of them can be implemented practically [20,21,22,23,24,25].

In the simplest case of homopolymer macromolecules, in a good solvent, the polymer layer will create a protective shell of swollen macromolecules (“hairy nanoparticles”) [17,26,27,28]; in a poor solvent, it will collapse, and, depending on the grafting density, completely or partially cover the nanoparticle surface, forming patchy patterns [12,15,17,28,29].

Grafted layers, made from different macromolecules and/or copolymer chains, self-assemble in very different structures. The type of structures depends on many additional parameters, including the compatibility of various monomer units with each other and solvent. In general, they aggregate in such a way that more soluble macromolecules/monomer units are exposed to the solvent, and less soluble units are maximally protected from contact with it [10,30,31].

If polymer blocks of different nature are combined into a comb-like copolymer, it can be expected that in the grafted layer they self-organize into cylindrical strands. Another possible option may be a striped, lamellar structure diverting from the grafting surface. Both expected structures—cylindrical strands and striped morphology—were experimentally observed in [32,33,34]. The amphiphilic comb-like copolymers, reported in [32,34], were designed via interpolymer complexation of polyanionic brushes with polycation-b-PEO copolymers. These comb-copolymers have hydrophobic (polyanion-polycation) backbone and hydrophilic PEO pendants. The self-assembly to microphase-separated structures allows to hide hydrophobic backbone and to increase the contact of PEO with solvent.

The divergent cylindrical strands and lamellas were reported also for the spherical nanoparticles coated with amphiphilic homopolymers [35,36,37]. The structures are referred to as hedgehogs and multipetalers, correspondingly. These figurative names were chosen because the lamellas on the surface fold akin to petals in buds; and concentrated solutions of nanoparticles with cylindrical aggregates resemble a solution of sea urchins. Notably, other cases of hedgehog and urchin structures with spikes of very different nature are also described in the literature [38,39,40].

The amphiphilic homopolymers are macromolecules composed of identical monomer units each containing both hydrophobic and hydrophilic groups [41,42,43,44]. The effective coarse-grained model represents such monomer units as a dumb-bell of two beads [41]. The dumb-bell monomer units are connected into chains such that some beads form backbone and the others are pendant groups. Additionally, amphiphilic homopolymers can thus be treated as a limiting case of comb-like copolymer with short side chains [45].

Firstly, the cylindrical strands and lamellas morphology are addressed for amphiphilic homopolymers tightly grafted in a flat surface [46]. The strands, composed of few macromolecules, are arranged perpendicular to the surface in hexagonal order and observed in the solvent being selectively good for pendants and poor for mainchains. The lamellas are expected in mirror-inverse case, with the solvent being poor for pendants and good for backbone. The lamellae are oriented perpendicular to the flat surface and are characterized by two slightly different periods [47]. The transitions between the lamellas with different periods occur through an intermediate (“parking-garage”) structure when lamellae with different periods coexist and are spaced in height.

Similarly, it can be assumed that in the case when amphiphilic macromolecules are grafted to spherical surface, the macromolecules are preferably assembled into cylindrical branches in the solvent, being selectively good for pendants and poor for mainchains (“hedgehogs”), and they are collected into lamellae in mirror-inverse case (“multipetalers”). Under these assumptions, the analytical theories were developed for each of these cases separately and results were presented in the form of state diagrams indicating the fields with different numbers of spikes and petals for hedgehogs and multipetalers, correspondingly. In the latter case, the data were confirmed by specially designed computer experiments [37].

The difference in morphologies observed in mirror-inverse solvents can be explained by the dissimilarity in entropy constraints during clustering of pendant and mainchain groups. With clustering caused by attraction between the side groups, the loss in entropy is much greater, due to significant restrictions imposed on the mobility of the pendants [48]. The restrictions favor the cluster fusion in poor for pendant solvent and lead to the effecting orientation-induced attraction. The orientation-induced attraction is purely entropic in nature, and its impact has made the cylindrical branches almost unprofitable for such solvent.

In solvent, being selectively good for pendant and poor for mainchains, the orientation-induced attraction is negligible, and it can be expected that both cylindrical and lamellae structures can be stable within noticeable regions.

The aim of this study is to develop a theoretical approach that allows us to describe various structures formed during self-assembly of amphiphilic homopolymers grafted to a spherical nanoparticle and to outline the areas of their stability. We examine the previously described hedgehog and the multipetal structures, and introduce the chamomile structure. In chamomile, the cylindrical branches are bent and connected at the ends. We propose a theoretical model considering the conformation and energy of individual macromolecules, taking into account the structures of individual aggregates and of their interaction with each other. This allows us to describe in detail the morphology of these complex structures as function of nanoparticle size, grafting density, polymerization degree, affinity of different groups to solvent and each other.

## 2. Model

In accordance with the main objective of the article, we will consider a decorated nanoparticle covered with a tightly grafted layer of an amphiphilic homopolymer.

**Figure 1:**
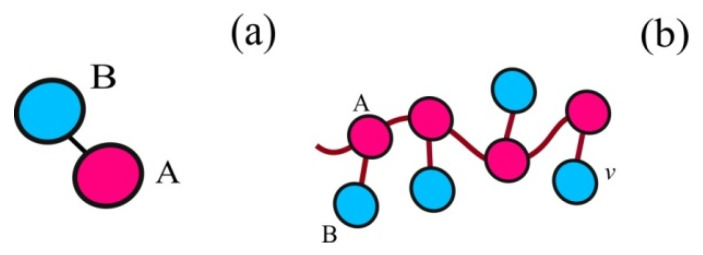
Model of a dimer unit (**a**) and amphiphilic homopolymer chain (**b**).

Let the nanoparticle be an impenetrable sphere with radius *R*, and the grafted layer consists of *M* polymer chains with a degree of polymerization *N*, *N* >> 1. The polymer chains are long, flexible and obey Gaussian statistics [49]. They consist of identical monomeric units, each containing groups with different affinity to the solvent. This is taken into account by presenting monomer units in the form of *A*-*graft*-*B* dumb-bells made of two *A* and *B* beads, with the same volumes *v* and different affinity to the solvent (Figure 1a). *A* beads are interconnected into chain backbone, *B* beads are pendants (Figure 1b). The hairy nanoparticle is immersed into selective solvent, being poor for *A* backbones and good for *B* pendants (Figure 1b). Let *v_s_* be volume of a solvent molecule: *v_s_* = *v*; *ε_Bs_* be the interaction energy of side *B* groups with solvent, *ε_Bs_* < 0; *ε_AA_* be the interaction energy of hydrophobic groups, *ε_AA_* < 0. The interaction energy parameters are expressed in the units of *kT* and account for the affinity of side *B* beads to solvent and effective attraction of *A* groups, which avoid contacts with solvent and pendant groups. In selective solvent, the grafted macromolecules combine with each other into complex morphologies in such a way that groups *A* are protected from contact with the solvent, and groups *B* are exposed to it as much as possible.

**Figure 2:**
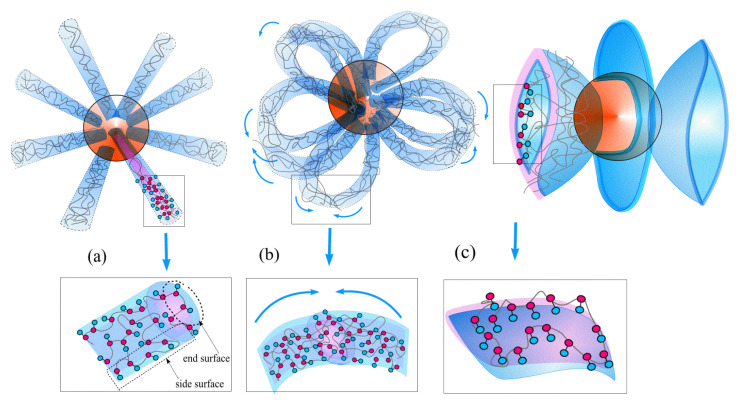
Schematic representation of hedgehog (**a**), chamomile (**b**), multipetal structure (**c**).

We have identified the most characteristic morphologies and will determine the areas of their stability. They are shown in Figure 2. These are the so-called hedgehogs [35], when macromolecules combine into spikes radially diverging from the nanoparticle (Figure 2a); chamomile, when these spikes unite at the ends and form loops (Figure 2b) and multipetalers [36], when macromolecules combine into thin membrane-like lamellae arranged in a symmetrical manner (Figure 2c). The depicted structures are very different, but what they have in common are that (*i*) grafted macromolecules are joined into several basic components (spike, loop or petal); (*ii*) the basic components have solvophilic surface from B beads; A beads are hidden inside; (*iii*) the volume fraction *φ* of monomer units inside basic component (spike, loop or petal) is high, close to unity: *φ*~1; (*iv*) the basic components can stretch, curve and interact with next to components (either spike, loop or membrane-like petal) with excluded volume repulsion and van der Waals attraction [49,50,51,52].

In that way, the free energy of the multi-component structure has to include contributions describing macromolecular interaction and conformation, surface free energy and those for elasticity and interaction of basic components; thus, it is determined by the complete morphological pattern on the surface of the decorated nanoparticle.

The free energies of elasticity of the basic components (spikes, loops or membrane-like petals) and their steric interactions were addressed in Helfrich approximation [50,51,52] adapted to describe core–shell structures of amphiphilic homopolymers [36,53]. For each of the structures (hedgehog, chamomile, multipetaler), all Helfrich terms were written out; then, their contributions were evaluated and only the significant ones were left. In paper, we have excluded the intermediate stages and present the resulting expressions for free energies.

In the following items, we consider each of the described morphologies consistently, propose a more specific and detailed model, write down the free energies and a way to minimize them.

## 3. Free Energy

### 3.1. Hedgehog

According to the above, in the hedgehog, the macromolecules combine into spike-like aggregates, radially diverging from the surface of the nanoparticle. Let *K* be the total number of spikes and assume that each spike has a cylindrical shape with length *L*, cross —section radius *r* and volume fraction of polymer *φ* (determined above).

The total free energy of hedgehog structure *f_h_* is a sum of the mixing free energy *f_int.h_*, the surface free energy *f_surf.h_*, the free energy of spike bending *f_curv.h_*, and that of spike’ steric interaction *f_ster.h_*:

(1) $$f_{h}=f_{\mathrm{int}.h}+f_{surf.h}+f_{curv.h}+f_{ster.h}$$

where all contributions are normalized on the total number of monomer units *MN* and expressed in *k_B_T* units.

The mixing free energy *f_int.h_* accounts for the pair interactions and translational entropy of solvent molecules [49]:

(2) $$f_{\mathrm{int}.h}=\frac{\varepsilon }{2}\varphi +\frac{v}{v_{s}}\left(\frac{1-\varphi }{\varphi }\ln(1-\varphi )\right)$$

where $\varepsilon =\varepsilon_{AA}-2\varepsilon_{Bs}<0$ is the effective interaction energy; and a coil conformation is taken as a reference state.

The surface free energy can be accounted for within the approximation generally accepted for such systems. It is written as the interaction energy of monomer units in thin, one-monomer units-thickness layer [54] and accounts for the differences in the structure of such layers on the side face and the end of the spike (Figure 2a):

(3) $$f_{surf.h}(\varphi )=\frac{K}{NM}(M_{side}\sigma_{side}+M_{end}\sigma_{end})$$

where *M_i_* and *σ_i_* are the total number of monomer units and the energy benefit for monomer units being within the near-surface layers at the corresponding part of spike (*i* = *side* and *end*).

$M_{i}\approx \varphi S_{i}/v^{2/3},$ where *S_i_* is surface area: $S_{side}=2\pi rL$ (*i* = *side*) and $S_{end}=\pi r^{2}$ (*i* = *end*).

On side surface, the protective shell consists only of B groups (Figure 2a) and $\sigma_{side}\sim \varepsilon_{Bs}\varphi /2$ [54]. The end surface is made of both A and B groups (Figure 2a), and the energy benefit per one monomer units can be estimated as $\sigma_{end}\sim \varepsilon \varphi /2$.

Given the above and that the volume of spike is $V=\pi r^{2}L$*,* the surface free energy per one monomer unit is rewritten as:

(4) $$f_{surf.h}(\varphi )=\varepsilon_{Bs}\varphi \frac{v^{1/3}}{r}+\frac{\varepsilon }{2}\frac{v^{1/3}}{L\varphi }$$

The Helfrich free energy of spike bending can be written as (see Supplementary Materials):

(5) $$f_{curv.h}(\varphi )=\frac{v}{r^{2}\varphi }\left(\frac{k_{1}}{v^{1/3}}+\frac{k_{2}}{r}\right)$$

where *k_1_* and *k_2_* are spontaneous and mean bending moduli, correspondingly.

The free energy of spike steric interaction reads as:

(6) $$f_{ster.h}(\varphi )=\frac{3\pi^{3}K^{2}r}{4Rk_{c}NM}$$

where *k_c_* is dimensionless elastic modulus.

It was obtained by the summarizing of the Helfrich free energy of the interaction between the neighboring spikes (see Supplementary Materials).

### 3.2. Chamomile

In chamomile, the nearest spikes are connected by their end surfaces, forming *K*/2 loops (Figure 2b). The loops have cross-section radius *r* and volume fraction of the polymer inside *φ*.

The free energy *f_c_* of chamomile structure, normalized on the total number of monomer units *NM* and temperature, is sum of four contributions:

(7) $$f_{c}=f_{\mathrm{int}.c}+f_{surf.c}+f_{curv.c}+f_{ster.c}$$

where *f_int.c_* describes the polymer-solvent interaction and is given by Equation (2).

The surface free energy *f_surf.c_*, taken within approximation described above, reads as:

(8) $$f_{surf.c}(\varphi )=\varepsilon_{Bs}\varphi \frac{v^{1/3}}{r}$$

The bending energy *f_curv.c_* accounts for that in the loop the cylindrical aggregates are in bending state with the curvature $C_{2}≅2\sqrt{1-4R^{2}/L^{2}K}/L$ (see Supplementary Materials) and reads as:

(9) $$f_{curv.c}(\varphi )=\frac{4k_{1}v^{2/3}}{\varphi r^{2}}+\frac{8\pi K}{NM}\left(\frac{k_{1}r}{v^{1/3}}+k_{G}\right)+\frac{4k_{2}v}{\varphi r^{3}}$$

where *k_1_*, *k_2_* and *k_G_* are the spontaneous, mean and Gaussian bending moduli.

The free energy of steric interaction *f_surf.c_* is slightly different from one, obtained for spikes (see Supplementary Materials); here, we use the same expression given in Equation (6).

### 3.3. Multipetal Structure

In the multipetal structure polymer chains are arranged into *K* thin flat petals of thickness Δ interacting with each other. The bending energy of flat bilayers is negligible compared with the free energy of their interaction with each other [35]; thus, free energy *f_m_* of the multipetal structure (per monomer unit) is the sum of three contributions: mixing free energy *f_int.m_*; surface free energy *f_surf.m_*, and Helfrich intra-petals interaction *f_ster.m_*:

(10) $$f_{m}=f_{\mathrm{int}.m}+f_{surf.m}+f_{ster.m}$$

where the first term *f_int.m_* is given by Equation (2); and the following two terms are derived in the approximation of non-curved petals (see Supplementary Materials):

(11) $$f_{surf.m}(\varphi )=\varepsilon_{Bs}\varphi /\left(2\Delta \right)$$

(12) $$f_{ster.m}(\varphi )=\frac{3\pi }{2R^{2}k_{c}}K^{3}\ln\left(\frac{NMv^{2/3}}{\pi KR^{2}\varphi \Delta }+1\right)$$

Thus, the total free energies of hedgehog, chamomile and multipetal structures are written as functions of the number of basic structural components, *K*, their radius, *r* (for spikes and loops) or width Δ (for petals) and polymer volume fraction inside the corresponding basic components *φ*.

Next, we obtain the minimal values of the free energy for all structures and compare them with each other. The minimization parameters are the number *K* of basic components (spikes, loops or petals)*,* their characteristic size (cross-section radius *r*, or petal thickness Δ) and polymer volume fraction *φ*.

## 4. Equilibrium Parameters of the Structures and Free Energies Comparison

In this section, we report results of the free energy *f_i_* (*i* = *h*, *c*, *m*) minimization and discuss the dependence of equilibrium values of structure parameters (total number *K*_o,*i*_ of basic components, and their main characteristics *r_o,i_* and Δ*_o_*) on solvent quality and selectivity.

In all the cases, the minimization of the free energies *f_i_* (*i* = *h*, *c,* *m*) allows us to transform the free energies to the function of the only variable—volume fraction of polymer *φ* and derive analytical expression for the equilibrium values of structure parameters.

The results of calculations for each of the structures are presented below. The characteristics radii *r_o,i_* and width Δ*_o_* are normalized on bead size *v*^1/3^:

Hedgehog:

(13) $$f_{h}=\frac{\varepsilon }{2}\varphi +\frac{v}{v_{s}}\left(\frac{1-\varphi }{\varphi }\ln(1-\varphi )\right)-\frac{\varepsilon_{Bs}^{2}}{8k_{1}}\varphi^{2}-\frac{16\varepsilon^{2}}{3NM}\frac{R}{v^{1/3}}\frac{\varphi k_{c}}{{\left|\varepsilon_{Bs}\right|}^{3}}$$

(14) $$K_{oh}\approx \frac{4\varphi k_{1}k_{c}}{3\pi }\frac{R}{v^{1/3}}\frac{\left|\varepsilon \right|}{\left|\varepsilon_{Bs}\right|}\;r_{oh}=\frac{4k_{1}}{\left|\varepsilon_{Bs}\right|\varphi }$$

Chamomile:

(15) $$f_{c}=\frac{\varepsilon }{2}\varphi +\frac{v}{v_{s}}\left(\frac{1-\varphi }{\varphi }\ln(1-\varphi )\right)-\frac{\varepsilon_{Bs}^{2}\varphi^{2}}{8k_{1}}-\frac{32k_{c}R}{{\left(NM\right)}^{3}v^{1/3}}\left(k_{1}+k_{G}\varepsilon_{Bs}\right)\left|k_{G}\right|$$

(16) $$K_{oc}\approx \frac{8k_{c}R}{3\pi NMk_{1}v^{1/3}}\left|\varepsilon_{Bs}\right|\varphi \left(\left|k_{G}\right|-\frac{4k_{1}^{2}}{\left|\varepsilon_{Bs}\right|\varphi }\right)\;r_{oc}=\frac{4k_{1}}{\left|\varepsilon_{Bs}\right|\varphi +\frac{16k_{c}R}{{\left(NM\right)}^{3}v^{1/3}}k_{G}^{2}}$$

Multipetal structure:

(17) $$f_{m}=\frac{\varepsilon }{2}\varphi +\frac{v}{v_{s}}\left(\frac{1-\varphi }{\varphi }\ln(1-\varphi )\right)-\frac{2\varphi^{3}R^{3}}{vNM}\sqrt{\frac{1}{3}\varepsilon_{Bs}^{2}k_{c}}$$

(18) $$\Delta_{o}\approx \frac{4NM}{3\pi \varphi^{2}\sqrt{2k_{c}\left|\varepsilon_{Bs}\right|}}\frac{v}{R^{3}}\;K_{om}\approx \sqrt{2k_{c}\left|\varepsilon_{Bs}\right|}\varphi \frac{R}{v^{1/3}}$$

where *k*_c_, *k*_1_ and *k_G_* are the elastic spontaneous, mean and Gaussian *k_G_* bending moduli, respectively (*k*_c_ > 0; *k*_1_ *>* 0; *k_G_* < 0). They are determined by the surface of basic components and depend on solvent-B interaction parameter *ε_Bs_* [53,55]. In this article, the calculations are performed at: *k*_c_ = *k*_1_ = −*ε_Bs_* and *k_G_* = −5|*ε_Bs_*|.

The expressions are obtained under the natural assumption that the nanoparticle radius is much larger the cross-section of aggregates (*R >> r_i_,* Δ). The details of calculation for each case are reported in Supplementary Materials.

Thus, for each set of *ε_AA_* and *ε_Bs_*, characterizing solvent quality, it is possible to estimate the minimum of free energy, and then to determine the resulting structure.

**Figure 3:**
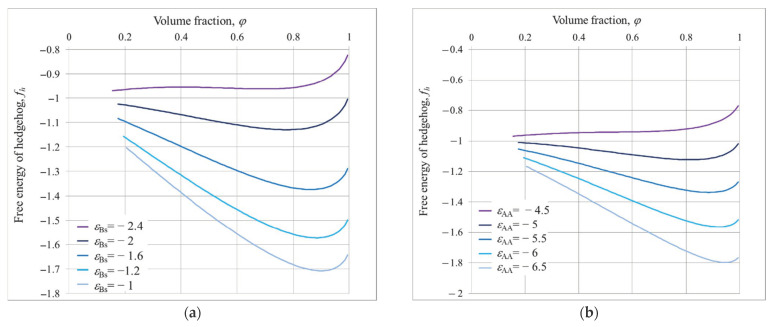
Free energy of hedgehog structure *f_h_* on dependence of volume fraction *φ* at different values of *ε_Bs_* and *ε_AA_* = −5 (**a**); different values of *ε_AA_* and *ε_Bs_* = −2 and (**b**); *R/v*^1/3^ = 10.

**Figure 4:**
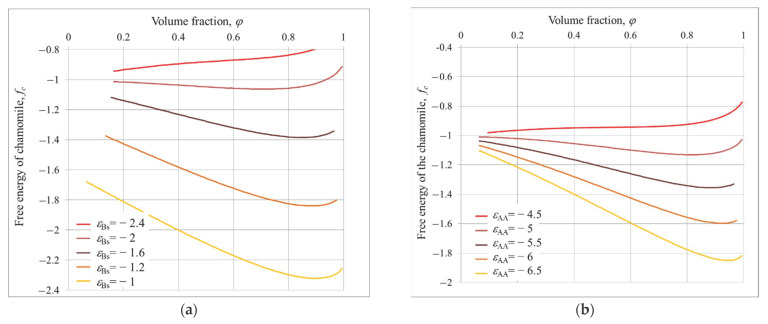
Free energy of chamomile structure *f_c_* on dependence of *φ* for different values of *ε_Bs_*, *ε_AA_* = −5 (**a**) and different values of *ε_AA_*, *ε_Bs_* = −2 (**b**); *R/v*^1/3^ = 10.

**Figure 5:**
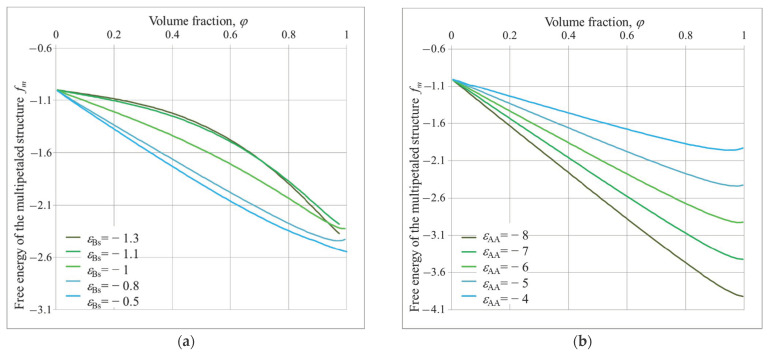
Free energy of multipetal structure *f_m_* as function of *φ* at fixed *ε_AA_* = −5 and different *ε_Bs_* (**a**); at fixed *ε_Bs_* = −1 and different *ε_AA_* (**b**), *R/v*^1/3^ = 10.

Examples of the dependences of free energy on the polymer volume fraction *φ* for different *ε_AA_* and *ε_Bs_* values and different possible structures are shown in Figure 3, Figure 4 and Figure 5.

The dependencies of hedgehog free energy *f_h_* are presented in Figure 3.

It is seen that in all the cases the free energy is nonmonotonic function with minimum at rather high *φ*. Such a minimum at large *φ* indicates the presence of stable hedgehog structures. At *ε_AA_* = −5 and *ε_Bs_* = −1 (Figure 3a), the minimum free energy falls on *φ_min_*~0.9. Additionally, the structure parameters calculated using Equation (14) are as follows: the total number of spikes *K_oh_* = 11 and their radius *r_oh_* = 4.4. With increasing the affinity of B groups to the solvent |*ε_Bs_*|, the minimum of free energy shifts to the lower *φ* values, the total number of spikes increases and their radii become smaller: at *ε_Bs_* = −1.2, *K_oh_* = 13 and *r_o_* = 3.7; and at *ε_Bs_* = −2, *K_oh_* = 14 and *r_o_* = 2.2 (Figure 3a).

At *ε_Bs_* = −2.4, the profile of free energy has two minima, and lower one is at *φ_min_*~0.01. It means that the grafted macromolecules do not aggregate; they swell freely. The transition from hedgehog to the uniform shell of swollen chains proceeds sharply within narrow intervals around *ε_Bs_*~−2.4, *φ*~0.7, *K_oh_* = 16.

The hedgehog structure, observed at *ε_Bs_*~−2 and *ε_AA_* = −5 (*φ_min_*~0.7, *K_oh_* = 14), is destroyed with decrease in A-A attraction (Figure 3b). It is seen that at *ε_AA_* = −4.5, the minimum of free energy is at *φ*~0.01. However, the profile of free energy now exhibits the only minimum: it indicates smooth hedgehog–coiled shell transition. Increase in A-A attraction (growth of absolute *ε_AA_* values) calls shifts of free energy minima to the high values and to disintegration and compaction of spikes.

Figure 4 shows the dependencies of chamomile free energy *f_c_* on polymer volume fraction *φ*.

One can see that the chamomile also can be stable within the same interval of solvent quality parameters: the free energies have minimum at *φ* *>>* 0.

At *ε_Bs_* = −1 and *ε_AA_* = −5 (Figure 4a) the minimum free energy is at *φ_min_*~0.88 and *K_oc_* = 3 and *r_oc_* = 4.2. With increasing |*ε_Bs_*|, i.e., affinity of B groups to the solvent, the minimum of free energy shifts to the lower *φ* values, the total numbers of loops increases and their radii become smaller: *ε_Bs_* = −1.2, *φ_min_*~0.89, *K_oc_* = 4 and *r_oc_* = 3.7; *ε_Bs_* = −1.6, *φ_min_*~0.85, *K_oc_* = 6 and *r_oc_* = 3; and *ε_Bs_* = −2, *φ_min_*~0.7, *K_oc_* = 8 and *r_oc_* = 2. At *ε_Bs_* = −2.4, the macromolecules do not aggregate (*φ_min_*~0.01) and make a swollen shell around nanoparticle.

Similarly to a hedgehog, the chamomile is destroyed when attraction A-A decreases, i.e., when the quality of the solvent for A groups improves, the total number of loops increases and they become thinner with worsening solvent quality for A groups (Figure 4a).

In Figure 5, the dependencies of multipetaler free energy *f_m_* on *φ* are shown.

At *ε_AA_* = −5 (Figure 5a), *f_m_* (*φ*) are nonmonotonic functions, having a minimum at *φ* ≤ 1, within a narrow *ε_Bs_* interval: −1 ≤ *ε_Bs_* ≤ −0.5. At *ε_Bs_* < −1, *f_m_* (*φ*) are convex functions. In this region, other structures – hedgehog or chamomile – are energetically favorable. At *ε_Bs_* > −0.5, the free energy drops to minimum values outside valuable interval: *φ* > 1, and grafted macromolecules are uniformly compacted around nanoparticles (compacted brush).

It is seen that the multipetal structure can be realized at much lower affinity of B groups to solvent and at stronger A-A attraction than hedgehog and chamomile (sf. Figure 3, Figure 4 and Figure 5). Additionally, the petals are much denser than the hedgehog spike and chamomile loops. The volume fraction of polymer *φ* within petal varies from *φ_min_*~0.88 up to *φ*~1. Correspondingly, the total number of petals varies from *K*= 5 (*ε_Bs_* = −0.8; *ε_AA_* = −5) to *K* = 10 (*ε_Bs_* = −1.1; *ε_AA_* = −5); and their width Δ*_o_*, measured in number of monomers, changes from Δ*_o_* = 15 to 4.

At fixed affinity of B groups to solvent (*ε_Bs_* = −1, Figure 5b), the growth of A-A attraction |*ε_AA_*|, i.e., worsening of solvent quality for A groups, calls shift of the free energy minima to larger value of *φ* up to *φ_min_*~1. Within this interval the total number of petals increases from *K* = 7 (*ε_AA_* = −4) to *K* = 10 (*ε_AA_* = −8).

**Figure 6:**
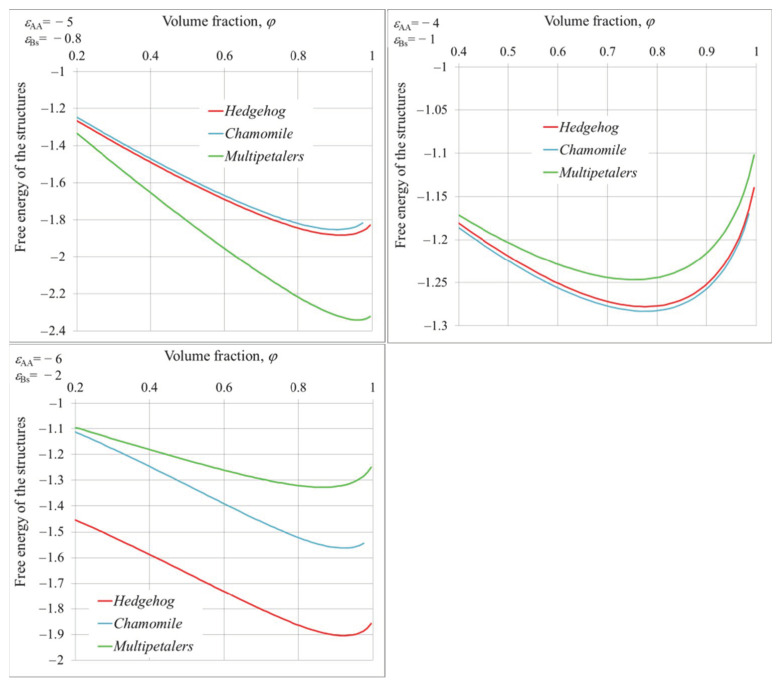
Relative position of free energy profiles for hedgehog, chamomile, and multipetal structures. *R/v*^1/3^ =10; *ε_AA_* and *ε_Bs_* are indicated in the insets.

Figure 6 presents the dependencies of free energies on volume fraction of polymer for various structures, calculated at fixed values of solvent parameters. Comparing the relative positions of the graphs and their minimum values, we can draw the following conclusions. With *ε_AA_* = −5, *ε_Bs_* = −0.8, multipetalers are the most preferable; at *ε_AA_* = −4, *ε_Bs_* = −1, the chamomiles have smallest free energy; and the hedgehogs can be detected at *ε_AA_* = −6, *ε_Bs_* = −2.

Consistently applying this procedure for different sets of parameters, we determined the stability regions of various structures and outlined their borders as lines with equal minima of free energies of neighboring structures. The results are summarized in the form of state diagrams.

## 5. State Diagrams

**Figure 7:**
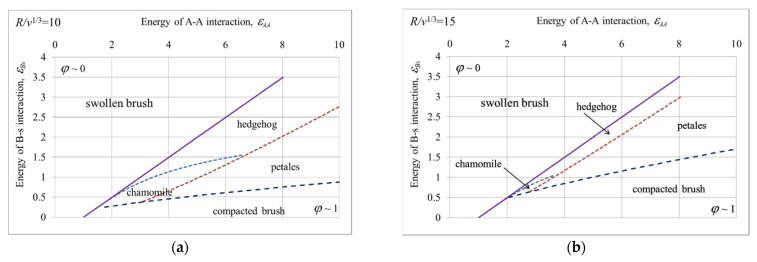
Structure diagram in variables |*ε_AA_*|, |*ε_Bs_*|, *R/v*^1/3^ = 10 (**a**), *R/v*^1/3^ = 15 (**b**), *N* = 100, *M* = 100.

In Figure 7, the state diagrams in variables |*ε_AA_*| and |*ε_Bs_*| are presented for different radii of nanoparticle *R* and the same total number *M* of grafted macromolecules and their degree of polymerization *N*.

The diagram highlights the area with hedgehogs, chamomiles, and multipetal structures. Additionally, the regions of swollen and compacted brushes are distinguished. Within these regions, the grafted macromolecules do not segregate and form either swollen or compacted homogeneous shells, correspondingly.

The position of the boundary between the swollen brush with coiled chains and the structured grafted layer was estimated by equating the effective interaction parameter to zero: *χ* = *ε* + *v/v_s_ =* 0. At *χ* > 0 the cumulative second virial coefficient is positive, and attraction between A groups is not strong enough for them to aggregate. The borderline between swollen coil brush and structured brushes is shown by thick violet line. The borderline of compacted brush is shown by a dark blue dash line. It has been defined numerically as an edge state, starting from which the minimum of free energy goes out of the physically significant interval of *φ*: *φ* ≥ 1 (an example is given above, see Figure 5a and discussion).

The grafted macromolecules stay in swollen state when both the attraction between A-A units and affinity B groups to solvent are weak (Figure 7a,b). At high A-A attractions, grafted macromolecules either self-assemble to different structure or collapse on the surface of the nanoparticles. The homogeneously compacted brushes are observed when the affinity of B groups to solvent is negligibly small. Noticeable values of |*ε_Bs_*| lead to the brush structuring; its growth favors the formation of structures with larger surface and calls the transition to multipetal, chamomile, hedgehog structures. At high |*ε_Bs_*| values, the grafted macromolecules preserve swollen state even at very strong A-A attraction. It is seen that the chamomiles have the smallest area in state diagrams, placed at intermediate values of A-A attraction and rather weak B-s affinity. At *R/v*^1/3^ = 15, the chamomile area is almost indiscernible (Figure 7b).

Possible sequences of morphological transitions with worsening solvent quality for A-A groups are as follows. At a significant affinity of B groups to solvent (|*ε_Bs_*| > 1.5 for *R/v*^1/3^ = 10 and |*ε_Bs_*| > 1 for *R/v*^1/3^ = 15), swollen brush transforms to hedgehog and then to multipetal structure. At weaker B-s affinity, the sequences of morphological transformations include also chamomile structure which can be intermediate state between hedgehog and multipetaler or follow right after the swollen brush. A significant part of the diagram is occupied by the area of a compact unstructured shell. Such a uniformly compact shell can be observed with a slight affinity of B groups and solvent. It is formed either as a result of the fusion of the petals of a multipetal structure (|*ε_Bs_*| > 0.25 for *R/v*^1/3^ = 10 and |*ε_Bs_*| > 0.75 for *R/v*^1/3^ = 15), or as a result of the collapse of a uniformly swollen shell (|*ε_Bs_*| < 0.25 for *R/v*^1/3^ = 10 and |*ε_Bs_*| < 0.75 for *R/v*^1/3^ = 15).

A cascade of morphological transitions can also be triggered by fixing |*ε_AA_*| and changing parameter |*ε_Bs_*| characterizing affinity of B groups to solvent. It is seen that the most complete set of possible states is realized at intermediate fixed values |*ε_AA_*|.

**Figure 8:**
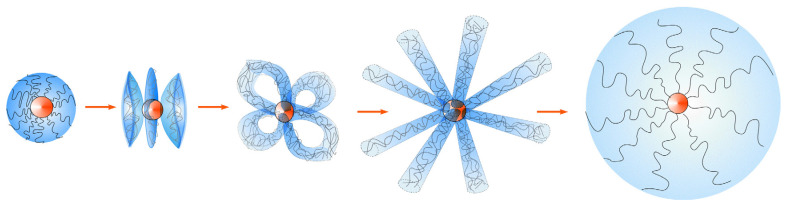
Sequence of structural transitions, observed at fixed *ε_AA_* = −5, *R/v*^1/3^ = 10 with an increase in B-s attractive energy: compacted brush—multipetaler—chamomile—hedgehog-swollen brush.

Schematic images of the sequence of morphological transformations for fixed *ε_AA_* and different |*ε_Bs_*| are shown in Figure 8. At small |*ε_Bs_*|, the grafted macromolecules are compacted; then, the compacted layer splits first into four dense petals, and then into seven petals. Further increase in |*ε_Bs_*| makes more stable chamomile structure with three and then with six loops. Then, six chamomile’s loops divide, and hedgehog with twelve and then with sixteen spikes is formed. Finally, with strong B-s affinity (large |*ε_Bs_*|), the macromolecules disaggregate, and swollen shell is formed around nanoparticle.

Figure 7a,b, were calculated for different nanoparticle radius *R* and the same total number *M* of grafted macromolecules and their degree of polymerization *N*. Thus, the nanoparticles differ in grafting densities of macromolecules: *ρ* *= M*/*R^2^*. It is extremely high (*ρ*~1) for *R/v*^1/3^ = 10, and twofold lower (*ρ*~0.44) for *R/v*^1/3^ = 15. It is with a decrease in the density of grafting chains that we associate with a reduction in the chamomile area. Rarely grafted chains, combined into rarely cylindrical aggregates, are unprofitable to bend to loops. Analysis shows that starting from *R/v*^1/3^ = 18 (*ρ*~0.3), one-hundred macromolecules (*M* = 100), each with one-hundred monomer units (*N* = 100), are unable to self-assemble into chamomile.

## 6. Conclusions

In this article, we proposed, for the first time, the generalizing theory of the self-organization of amphiphilic homopolymers grafted to a spherical surface. The amphiphilic homopolymers are macromolecules made of identical monomer units exhibiting groups with very different properties and affinity to solvents. We consider amphiphilic homopolymers within the framework of the commonly used coarse-grained two-beads A-graft-B model and assume that the main chain A beads are hydrophobic, and the side pendants B are hydrophilic. The amphiphilic structure of monomer units causes the self-assembly of macromolecules to cylindrical spikes, loops, and petals-like membrane, in which hydrophobic groups are collected in the inner part, and hydrophilic groups are exposed to the solvent. The basic aggregates (spikes, loops, and petals) are distributed in a complex way on the surface, forming hedgehog, chamomile, and multipetal structures. The theory accounts for both the properties of individual macromolecules (their energy and elasticity) and the properties of emerging aggregates (their bending and steric interactions). The latter were described within the framework of the Helfrich approximation, developed earlier for the surfaces made by amphiphilic monomer units. This approach allows us to determine the conditions for the emergence of such structures, to describe these structures in detail, and to outline the areas of their stability. The results are presented in the form of state diagrams and are in qualitative agreement with the computer modeling of amphiphilic homopolymers and experimental data on comb-like macromolecules grafted to nanoparticles.

It is necessary to emphasize the timeliness and relevance of this theoretical research nowadays, when more and more papers are devoted to the creation and application of nanoparticles with a structured and stimuli-sensitive surface.

## Data Availability

The data presented in this study are available on request from the corresponding author.

## Supplementary Materials

The following supporting information can be downloaded at: https://www.mdpi.com/article/10.3390/polym14204358/s1.
