# Supplementary material for: Hedgehog, Chamomile and Multipetal Polymeric Structures on the Nanoparticle Surface: Theoretical Insights
Source: Polymers (Basel). 2022 Oct 16;14(20):4358. doi: 10.3390/polym14204358 (PMC9609382; doi:10.3390/polym14204358)
Supplement: Supplementary file 1 [file polymers-14-04358-s001.zip › polymers-1964556-supplementary.pdf]

Supplementary Materials

for

"Hedgehog, chamomile and multipetal polymeric structures on the nanoparticle surface: theoretical insights" by A. S. Ushakova and V. V. Vasilevskaya

1. Helfrich energy of steric interaction between neighboring components

The free energy of steric interaction of neighboring basic components (spikes, loops, or petals) in Helfrich approximation is written as:

$$f_{ster}(\varphi)NM = \frac{3\pi^2}{k_c}\frac{K}{2}\left[\int_{S_{side}} \frac{dS_{side}}{\left(d(l)\right)^2}\right], \qquad \text{(S1)}$$

where $d_i(l)$ is distance between neighboring basic components (Fig. S1); and integral is taken over their surface S_{side}.

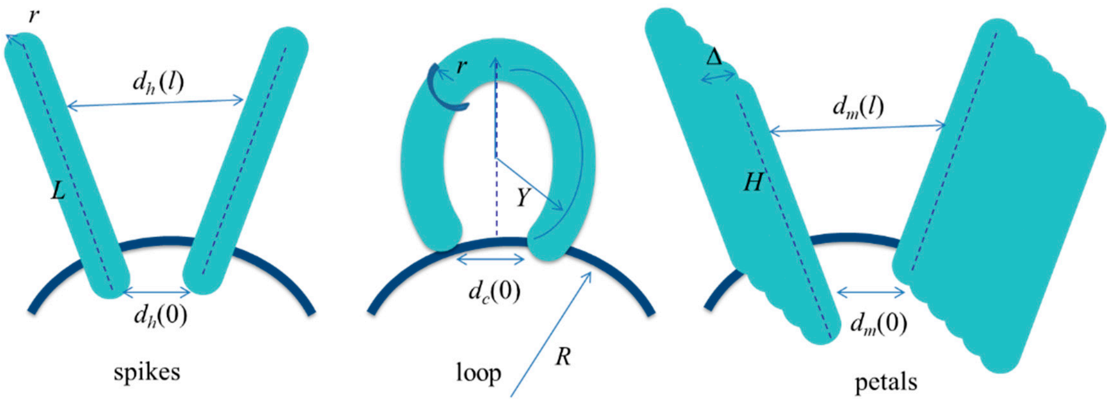

Figure S1. Schematic presentation of geometric relations: spike, loop and petal

Hedgehog

At uniform distribution of K spikes, the distance $d_H(l)$ between neighbors is:

$$d_h(l) = d_h(0)\left(R+L\right)/R, l = 0...L$$

where $d_h(0)$ *is* distance between spikes on the nanoparticle surface: $K\pi d(0)^2 = 4\pi R^2 - K\pi r^2$

$$d(0) = \sqrt{\frac{4R^2}{K} - r^2} + r \approx \sqrt{\frac{4R^2}{K}}$$

Helfrich free energy of interaction is:

$$f_{ster.h}(\varphi)NM = \frac{3\pi^2}{k_c}\frac{K^2}{2}\int_R^{R+L}\frac{2\pi r dl}{\left(R+l\right)^2} = \frac{3\pi^3}{k_c}\frac{K^2 rL}{4R(R+L)} \approx \frac{3\pi^3}{4k_c}\frac{K^2 r}{R}, L>>R. \qquad \text{(S2)}$$

Chamomile

Within the framework of the proposed model (see Fig. S1), up to numerical factors, the interaction energy $f_{ster.c}$ of chamomile loops at $L>>R$ coincides with the expression (S2)

Multipetal structure

In case of symmetric position of K petals relative to an arbitrary equatorial line, the distance between them is:

$$d_m(l) = d_m(0)\, l/R = 2\pi\, l/K\,,\; l = R...H$$

where $d_m(0)=2\pi R/K$ is distance between petals at nanoparticle surface.

Free energy $f_{ster.m}$ is

$$f_{ster.m}(\varphi)NM = \frac{6\pi^2}{k_c}\frac{K^3}{4\pi^2}\int_R^H \frac{2\pi z dz}{(l)^2} = \frac{3\pi}{2k_c}K^3 Ln\frac{H}{R}\,, \qquad \text{(S3)}$$

Taking into account that flat petals have surface area: $S_0 = \pi(H^2 - R^2) = \dfrac{NMv^{2/3}}{\pi K R^2 \varphi\Delta}$, where Δ is thickness, we obtain eq. (12).

2. Helfrich energy of aggregate bending

In general, the Helfrick free energy of bending is written as:

$$\frac{df_{curv}}{dS_{side}}NM = \frac{k_1}{v^{1/3}}\left(\frac{1}{R_1}+\frac{1}{R_2}\right) + k_2\left(\frac{1}{R^2_1}+\frac{1}{R^2_2}\right) + \frac{k_G}{R_1 R_2}\,, \qquad \text{(S4)}$$

where R_1 and R_2 are curvature radii; k_c, k_1 and k_G are elastic spontaneous, mean and Gaussian k_G bending moduli (k_c>0; k_1>0; k_G<0).

Hedgehog

For straight and long cylindrical spikes $R_1=r$ and $R_2=\infty$, and free energy of bending is:

$$\frac{df_{curv.h}}{dS_{side}}NM == \frac{k_1}{v^{1/3}r} + \frac{k_2}{r^2}\,,$$

$$\text{and } f_{curv.h}(\varphi) = \left(\frac{2k_1 v^{1/3}}{r} + \frac{k_2 v^{2/3}}{2r^2}\right)\frac{S_{side}}{NM} = \frac{v}{r^2\varphi}\left(\frac{2k_1}{v^{1/3}} + \frac{k_2}{r}\right) \qquad \text{(S5)}$$

Chamomile

Two characteristic curvatures of loop can be distinguished. The curvature of loop cross-section $R_1=r$ and curvature R_2 of loops as a whole: $Y = L/(2\sqrt{1-4R^2/L^2K})$

The surface area of loops is: $S_{side} = 2\pi r Y \cdot 2L/Y$, the bending free energy is:

$$f_{curv.c}NM = 4\pi K\left(\frac{k_1}{v^{1/3}}\left(L + 2r\sqrt{1-\frac{4R^2}{L^2K}}\right) + k_2\left(\frac{L}{r} + \frac{2r}{L}\left(1-\frac{4R^2}{L^2K}\right)\right) + 2k_G\sqrt{1-\frac{4R^2}{L^2K}}\right), \qquad \text{(S6)}$$

taken that $L=NMv/(K\varphi\pi r^2)$, and neglecting terms $\sim 1/N^2$ we obtain:

$$f_{curv.c}(\varphi) = \frac{4k_1 v^{2/3}}{\varphi r^2} + \frac{8\pi K}{NM}\left(\frac{k_1 r}{v^{1/3}} + k_G\right) + \frac{4k_2 v}{\varphi r^3}$$

Multipetal structure

With the accuracy adopted in these calculations, the free energy of the bending of the petals can be neglected [35].
